# Loading into Nanoparticles Improves Quercetin's Efficacy in Preventing Neuroinflammation Induced by Oxysterols

**DOI:** 10.1371/journal.pone.0096795

**Published:** 2014-05-06

**Authors:** Gabriella Testa, Paola Gamba, Ulya Badilli, Simona Gargiulo, Marco Maina, Tina Guina, Simone Calfapietra, Fiorella Biasi, Roberta Cavalli, Giuseppe Poli, Gabriella Leonarduzzi

**Affiliations:** 1 Department of Clinical and Biological Sciences, University of Torino, Orbassano, Italy; 2 Department of Pharmaceutical Technology, University of Ankara, Ankara, Turkey; 3 Department of Drug Science and Technology, University of Torino, Torino, Italy; Biological Research Centre of the Hungarian Academy of Sciences, Hungary

## Abstract

Chronic inflammatory events appear to play a fundamental role in Alzheimer's disease (AD)-related neuropathological changes, and to result in neuronal dysfunction and death. The inflammatory responses observed in the AD brain include activation and proliferation of glial cells, together with up-regulation of inflammatory mediators and of free radicals. Along with glial cells, neurons themselves can also react and contribute to neuroinflammatory changes in the AD brain, by serving as sources of inflammatory mediators. Because excess cholesterol cannot be degraded in the brain, it must be excreted from that organ as cholesterol oxidation products (oxysterols), in order to prevent its accumulation. Among risk factors for this neurodegenerative disease, a mechanistic link between altered cholesterol metabolism and AD has been suggested; oxysterols appear to be the missing linkers between the two, because of their neurotoxic effects. This study shows that 24-hydroxycholesterol, 27-hydroxycholesterol, and 7β-hydroxycholesterol, the three oxysterols potentially implicated in AD pathogenesis, induce some pro-inflammatory mediator expression in human neuroblastoma SH-SY5Y cells, via Toll-like receptor-4/cyclooxygenase-2/membrane bound prostaglandin E synthase (TLR4/COX-2/mPGES-1); this clearly indicates that oxysterols may promote neuroinflammatory changes in AD. To confirm this evidence, cells were incubated with the anti-inflammatory flavonoid quercetin; remarkably, its anti-inflammatory effects in SH-SY5Y cells were enhanced when it was loaded into β-cyclodextrin-dodecylcarbonate nanoparticles, versus cells pretreated with free quercetin. The goal of loading quercetin into nanoparticles was to improve its permeation across the blood-brain barrier into the brain, and its bioavailability to reach target cells. The findings show that this drug delivery system might be a new therapeutic strategy for preventing or reducing AD progression.

## Introduction

Alzheimer's disease (AD) is a progressive neurodegenerative disorder, typified by the pathological accumulation of β-amyloid (Aβ) peptides and neurofibrillary tangles (NFT) within the brain. It is the leading cause of dementia [1], [2]. Although the pathophysiology of AD remains poorly understood, considerable evidence indicates that multifactorial components participate in the progression of the disease, including inflammation, oxidative stress, altered cholesterol metabolism, glial cell activation, and dysregulation of intercellular communication among brain cells [3].

There is now mounting evidence to suggest that chronic inflammation plays a fundamental role in the progression of neuropathological changes in AD, resulting in neuronal dysfunction and death [4]–[8]. In this connection, there is also increasing evidence that central nervous system (CNS) and systemic inflammation cannot be viewed in isolation [9]. Even low grade systemic inflammation might have important CNS consequences in AD individuals, exacerbating behavioral symptoms and accelerating disease progression, due to the increased production of local pro-inflammatory cytokines and chemokines, as well as of reactive oxygen species (ROS) and nitric oxide (NO) [10], [11]. The detrimental effects of peripheral pro-inflammatory mediators in the AD brain chiefly occur because these agents enter the brain, together with infiltrating leukocytes, thanks to the increased blood-brain barrier (BBB) permeability as the disease progresses [12], [13].

The importance of neuroinflammatory processes has emerged from intensive study of the brain of AD patients. These have evidenced the activation and proliferation of microglia and astrocytes, together with enhanced release of neurotoxic cytokines, chemokines, complement components, inflammatory enzymes and acute phase proteins, as well as increased free radical-mediated oxidative stress [14]–[18]. However, it remains unclear whether inflammation is a cause or a consequence of AD. Clinical and experimental studies support the involvement of inflammatory changes already in the early stages of AD, even before the appearance of Aβ deposits. One of the main outcomes of microglia activation is thus the initiation of an innate immune response, dominated by the release of pro-inflammatory cytokines. Incidental to this are also phagocytosis of amyloid fibrils and large Aβ aggregates, suggesting an initial neuroprotective defense mechanism [19], [20]. In later stages of the disease, however, overproduction of inflammatory cytokines makes the microglia phagocytically inactive [21]. Along with glial cells, more recent evidence suggests that even neurons themselves react and contribute to the chronic neuroinflammatory changes in AD, by serving as source of inflammatory mediators [6].

The brain, the organ with the highest cholesterol concentration, cannot itself degrade cholesterol. Important mechanism whereby the brain eliminates excess cholesterol, in order to prevent its accumulation, is through the formation, and excretion into the circulation, of oxysterols, a class of cholesterol oxidation products which, unlike cholesterol, can easily cross the BBB [22], [23]. The major oxysterols involved in this excretion mechanism are 24-hydroxycholesterol (24-OH), and 27-hydroxycholesterol (27-OH). Of note, the marked accumulation of 27-OH in the AD brain is also due to an increased influx of this oxysterol across the BBB, because of hypercholesterolemia [24] or damaged BBB integrity [13]. A further compound, 7β-hydroxycholesterol (7β-OH), may also derive in the brain from oxidation of cholesterol, following the interaction of cholesterol with Aβ and amyloid precursor protein (APP) [25].

Regarding risk factors for AD, a growing body of epidemiological and molecular evidence suggests there may be a mechanistic link between altered brain cholesterol metabolism and AD, in which process oxysterols appear to be the missing linkers [22], [23], [26]–[28]. This idea has been supported by research pointing to the involvement of 24-OH and 27-OH in neurotoxicity, mainly by interacting with Aβ peptide [23].

Among therapeutic strategies that might successfully target ongoing brain inflammation during AD progression, dietary polyphenols, which can cross the BBB [29], have recently been proposed to play a role [30]–[35]. Among dietary polyphenols, the flavonoid quercetin is the most promising compound for AD prevention and therapy. Together with its ability to scavenge toxic free radicals such as ROS, this multipotent bioflavonoid can potentially reduce inflammatory processes [36]–[38]. To improve polyphenols' entrance into the brain, and their bioavailability and ability to reach the target tissue of interest for AD, new and particular delivery forms have been developed. One such method involves the use of nanoparticle carriers coupled to polyphenols [39]–[42]. This procedure applied to quercetin, for both oral and intravenous administration, has shown promising results [43], [44].

Since several oxysterols have been shown to elicit strong pro-inflammatory responses in a variety of cell types [45]–[50], we tested the hypothesis that 24-OH, 27-OH and 7β-OH might also promote inflammation in human neuroblastoma SH-SY5Y cells. It was hoped thus to provide clear evidence that oxysterols contribute to neuroinflammatory changes in AD. The findings demonstrate that these three oxysterols induce the expression of some inflammatory mediators in SH-SY5Y cells via Toll-like receptor-4/cyclooxygenase-2/membrane bound prostaglandin E synthase (TLR4/COX-2/mPGES-1). To highlight the inflammatory action of these oxysterols, the cells were then incubated with the bioflavonoid quercetin, which is known to possess strong anti-inflammatory properties. With the hope of finding a new delivery form for quercetin, to improve its bioavailability and consequently its anti-inflammatory activity, it was decided first to investigate, in an in vitro experimental study, this ability of quercetin's, when loaded into β-cyclodextrin (β-CD)-dodecylcarbonate nanoparticles. The anti-inflammatory effect of quercetin carried by nanoparticles was markedly enhanced versus that of free quercetin. This drug delivery system appears to be a potential new therapeutic approach, which might increase the neuroprotective effects of quercetin, improving both its permeation across the BBB into the brain, and its bioavailability to reach target cells.

## Materials and Methods

### Preparation and characterization of quercetin-β-CD-dodecylcarbonate inclusion complexes

The quercetin-β-CD-dodecylcarbonate inclusion complex was obtained by adding a suitable amount of quercetin to a dodecylcarbonate water/ethanol solution (75∶25 v/v) at a concentration of 10 mg/ml. This inclusion complex was prepared and characterized as described elsewhere for the alkylcarbonates of γ-cyclodextrin [51]. β-CD was a kind gift from Roquette (Lestrem, France). The amount of quercetin complexed was determined spectrophotometrically at 370 nm after dilution of a weighed amount of the complex in ethanol. After characterization, the complexes were used to prepare the nanoparticles.

### Preparation and characterization of coumarin 6-β-CD-dodecylcarbonate inclusion complexes

An excess of coumarin 6 (4 mg), as fluorescent marker, was added to β-CD dodecylcarbonate water/ethanol solution (75∶25 v/v) (10 mg/ml) to prepare fluorescent inclusion complexes. The suspension was left at room temperature in the dark for 5 days and then centrifuged. The supernatant was separated and freeze-dried to obtain the complex as powder form. The coumarin 6-β-CD-dodecylcarbonate inclusion complex was characterized by Differential Scanning Calorimetry [51] and by fluorescent spectroscopy (λ_ex_ = 450 nm and λ_em_ = 490 nm) using a Shimadzu RF-551 instrument.

### Preparation, characterization and in vitro release study of quercetin-loaded β-CD-dodecylcarbonate nanoparticles

Quercetin-loaded nanoparticles were prepared using dodecylcarbonates pre-loaded as a complex. Nanoparticles were obtained by the solvent injection technique as described elsewhere [52], [53]. Briefly the β-CD-dodecylcarbonate (20 mg) was dissolved in ethanol (3 ml); the solution was then added drop-wise to 20 ml water under stirring, to form β-CD-dodecylcarbonate based nanoparticles. After purification, nanoparticles were freeze-dried to obtain nanoparticles in powder form. Fluorescent nanoparticles were obtained with the same method using coumarin 6-β-CD-dodecylcarbonate inclusion complexes.

The average diameter and polydispersity index of nanoparticles were determined by Photon Correlation Spectroscopy, using a 90 PLUS instrument (Brookhaven, NY, USA) at a fixed angle of 90° and a temperature of 25°C. The electrophoretic mobility and zeta potential of nanoparticles were determined using a 90 Plus instrument (Brookhaven). The electrophoretic mobility measured was converted into zeta potential using the Smoluchowsky equation [54]. The nanoparticles' morphology was evaluated by Transmission Electron Microscopy using a Philips CM10 instrument (Eindoven, NL). The amount of quercetin incorporated into the nanoparticles was determined spectrophotometrically at 370 nm after dilution of a weighed amount of the complex in ethanol.

In vitro quercetin release experiments were carried out by the dialysis bag technique. A weighed amount of freeze-dried nanoparticles was dispersed in phosphate-buffered saline (PBS) pH 7.4 (2 ml), and placed in the donor compartment for 24 h. The receiving compartment was filled with a solution of 1% Tween 80/PBS pH 7.4 (50 ml). Withdrawn solutions were then analyzed spectrophotometrically to determine the concentration of quercetin.

### Cell viability assay

To test the cytotoxic effects of the β-CD-dodecylcarbonate nanoparticles alone or complexed with quercetin, cells were incubated with the compounds or left untreated for 48 h. After treatment, cell viability was measured in terms of the release of the enzyme lactate dehydrogenase (LDH). LDH activity was determined in culture medium using a photometrical assay based on the conversion of pyruvic acid to lactic acid by this enzyme, in the presence of reduced NADH. Control and nanoparticle supplemented cell values are expressed as percentages of total LDH released by untreated cells (100%), which were lysed with PBS plus 5% Triton X-100.

### Analysis of cell uptake of fluorescent nanoparticles by confocal laser microscopy

SH-SY5Y cells were grown on glass slides and, after treatments with coumarin 6-β-CD-dodecylcarbonate nanoparticles for the indicated times, specimens were washed (0.1 M PBS) and mounted with glycerol/distilled water (1∶1) plus 0.1% NaN_3_. Slides were observed through the LSM510 confocal laser microscope (Carl Zeiss SpA, Arese, Milan, Italy) equipped with an inverted microscope with Plan-NEOFLUAR lenses (40X/0.75).

### Cell culture and treatments

Human neuroblastoma SH-SY5Y cells were grown in RPMI 1640 medium containing 10% fetal bovine serum, 2 mM glutamine, 1% non-essential aminoacids and 1% antibiotic mixture (penicillin-streptomycin-amphotericin). Cells were incubated with 7β-OH, or 24-OH, or 27-OH (Steraloids, Newport, RI, USA), all at the non-cytotoxic final concentration of 5 µM, or with 15 µM oxysterol mixture (comprising 7β-OH, 24-OH, plus 27-OH, each present at the concentration of 5 µM), in all cases dissolved in ethanol (solvent). Untreated cells were taken as controls, and cells treated with 31.2 mM or 93.6 mM ethanol (equivalent concentrations of ethanol corresponding to 5 µM or 15 µM oxysterol mixture, respectively) as solvent controls. Cells supplemented with blank nanoparticles (not loaded with quercetin) or with blank nanoparticles (1 h pretreatment) plus oxysterols, were taken as internal controls. In experiments, some cells were pretreated (1 h) with 5 µM free quercetin (Q_F_) (Sigma-Aldrich, Milan, Italy) or with 5 µM quercetin loaded into nanoparticles (Q_N_) before oxysterol treatment. Incubation times for all experiments are reported in the Results section and Figure legends.

### RNA extraction and cDNA synthesis

Total RNA was extracted using TRIzol Reagent (Applied Biosystems, Monza, Italy) following the manufacturer's instructions. RNA was dissolved in RNase-free water fortified with RNase inhibitors (RNase SUPERase-In; Ambion, Austin, TX, USA). The amount and purity (A260/A280 ratio) of the extracted RNA were assessed spectrophotometrically. cDNA was synthesized by reverse transcription from 2 µg RNA with a commercial kit (High-Capacity cDNA Reverse Transcription Kit; Applied Biosystems) following the manufacturer's instructions.

### Real time RT-PCR

Singleplex real-time RT-PCR was performed on 30 ng of cDNA using TaqMan Gene Expression Assay kits prepared for human CD36, β1-integrin, interleukin 8 (IL-8), monocyte-chemoattractant protein-1 (MCP-1), matrix metalloproteinase-9 (MMP-9), TLR4, mPGES-1, and β_2_-microglobulin, TaqMan Fast Universal PCR Master Mix, and 7500 Fast Real-Time PCR System (Applied Biosystems). Negative controls did not include cDNA. The oligonucleotide sequences are not revealed by the manufacturer because of proprietary interests. The cycling parameters were as follows: 20 s at 95°C for AmpErase UNG activation, 3 s at 95°C for AmpliTaq Gold DNA polymerase activation, 40 cycles of 3 s at 95°C (melting), and 30 s at 60°C (annealing/extension). The fractional cycle number (Ct) at which fluorescence passes the threshold in the amplification plot of fluorescence signal versus cycle number was determined for each gene considered. The results were then normalized to the expression of β_2_-microglobulin, as housekeeping gene. β_2_-microglobulin was used as reference gene in all experiments, since preliminary observations had shown that expression of this housekeeping gene is not affected by oxysterol treatment. Relative quantification of target gene expression was achieved with a mathematical method [55].

### Western blotting

Whole-cell extracts were prepared in ice-cold lysing buffer (1 ml PBS) was added with 10 µl Triton X-100, 10 µl SDS 10%, 5 µl DTT 1 M, 6 µl PMSF 0.1%, 10 µl aprotinin). Total proteins (50 µg) were separated by electrophoresis in 10% denaturing SDS/polyacrylamide gel, then transferred to Hybond ECL nitrocellulose membrane (GE Healthcare Europe, Milan, Italy). After saturation of non-specific binding sites with 5% non-fat milk in Tris-buffered saline (TBS) 1x-Tween 20 0.05%, the membrane was immunoblotted overnight at 4°C with the primary antibody against COX-2 (1∶250) and subsequently probed with an anti-goat secondary antibody (1∶1000) (Santa Cruz Biotechnology Inc., Santa Cruz, CA, USA) overnight at 4°C. The membrane was stripped (Restore Western Blot Stripping buffer, Pierce Biotechnology, Rockford, IL, USA) and re-immunoblotted with anti-actin primary antibody (1∶10000) and then with anti-rabbit secondary antibodies (1∶7500) (Santa Cruz Biotechnology Inc.). The immunoreactive bands were visualized through enhanced chemiluminescence using the ECL-plus kit (GE Healthcare Europe) following the manufacturer's protocol. The bands were quantified by densitometric analysis using Image J 1.43 software. The results were evaluated as relative units determined by normalization of the density of each band to that of the corresponding actin protein band.

### Statistical analysis

All values are expressed as means ± SD. Data were analyzed statistically using one-way ANOVA with Bonferroni's post test for multiple comparisons. Differences at P<0.05 were considered statistically significant.

## Results

### 24-OH, 27-OH, and 7β-OH promote pro-inflammatory molecule expression in SH-SY5Y cells

**Figure 1:**
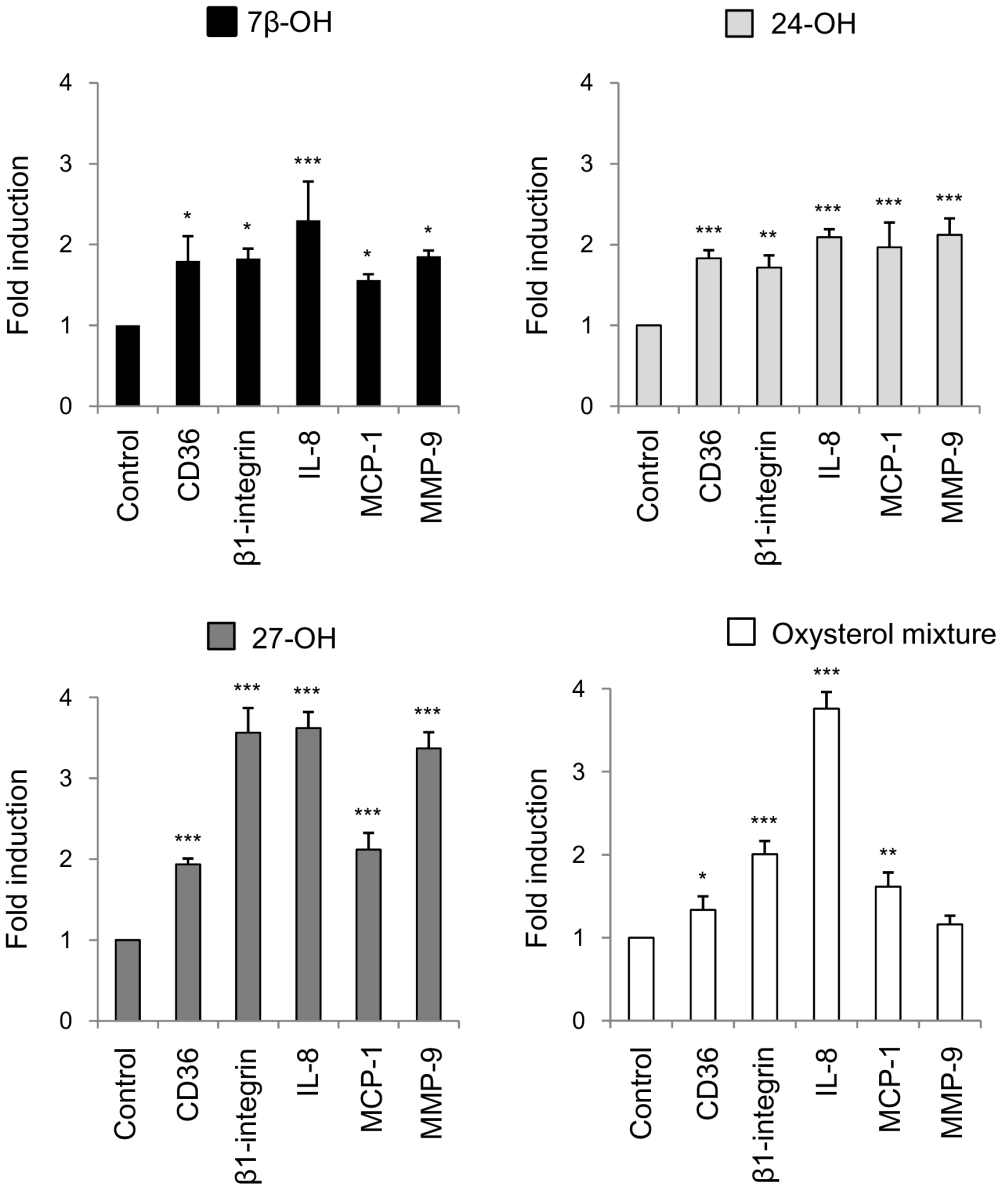
Effect of oxysterols on expression of CD36, β1-integrin, IL-8, MCP-1, and MMP-9. Gene expression was quantified by real-time RT-PCR in SH-SY5Y cells treated for 6 h with 5 µM 7β-hydroxycholesterol (7β-OH), 24-hydroxycholesterol (24-OH), 27-hydroxycholesterol (27-OH) or with a 15 µM mixture of these three oxysterols. Untreated cells (Control) were taken as controls. Data, normalized to β_2_-microglobulin, are expressed as mean values ± SD of three different experiments. *P<0.05, **P<0.01, and ***P<0.001 vs. control.

The three oxysterols that have been linked to the pathophysiology of AD, 24-OH, 27-OH, and 7β-OH, were first checked for their potential roles in modulating pro-inflammatory mediators in human neuroblastoma SH-SY5Y cells. In these cells, a marked and statistically-significant expression in various pro-inflammatory molecules was observed after 6 h cell incubation with any of the three oxysterols (5 µM) compared with untreated cells (controls). As Figure 1 shows, there were net increases of mRNA levels in chemokines IL-8 and MCP-1, the adhesion molecule β1-integrin, the scavenger receptor CD36, and MMP-9. Particularly, and in support of other reported findings, 24-OH and 27-OH appeared to be effective at promoting inflammatory response in neurons. The results were similar when cells were incubated with a mixture of the three oxysterols (15 µM) for 6 h: also in this case, expression of the inflammatory mediators considered was markedly increased in cells incubated with the oxysterol mixture, compared with controls, except for MMP-9 that was not significantly up-regulated (Figure 1). Cells treated with ethanol (solvent) remained unaffected (data not shown).

### Quercetin-loaded nanoparticles prevent the expression increase of inflammatory mediators induced by oxysterols in SH-SY5Y cells

The flavonoid quercetin, because of its anti-inflammatory effects, might be a promising candidate for preventing neuroinflammation in the brain. With the hope of finding a new delivery form for quercetin, to improve its permeation across the BBB into the brain and enhance its bioavailability, and thus also its therapeutic efficacy in AD, β-CD-dodecylcarbonate nanoparticles containing quercetin were formulated [see Materials and Methods]. To support the hypothesis that better brain delivery and bioavailability of quercetin would enhance its neuroprotective activity by preventing or reducing inflammatory changes in the brain, in our in vitro experimental model human neuroblastoma SH-SY5Y cells were preincubated either with 5 µM free quercetin (Q_F_) or with 5 µM quercetin loaded into β-CD-dodecylcarbonate nanoparticles (Q_N_).

**Figure 2:**
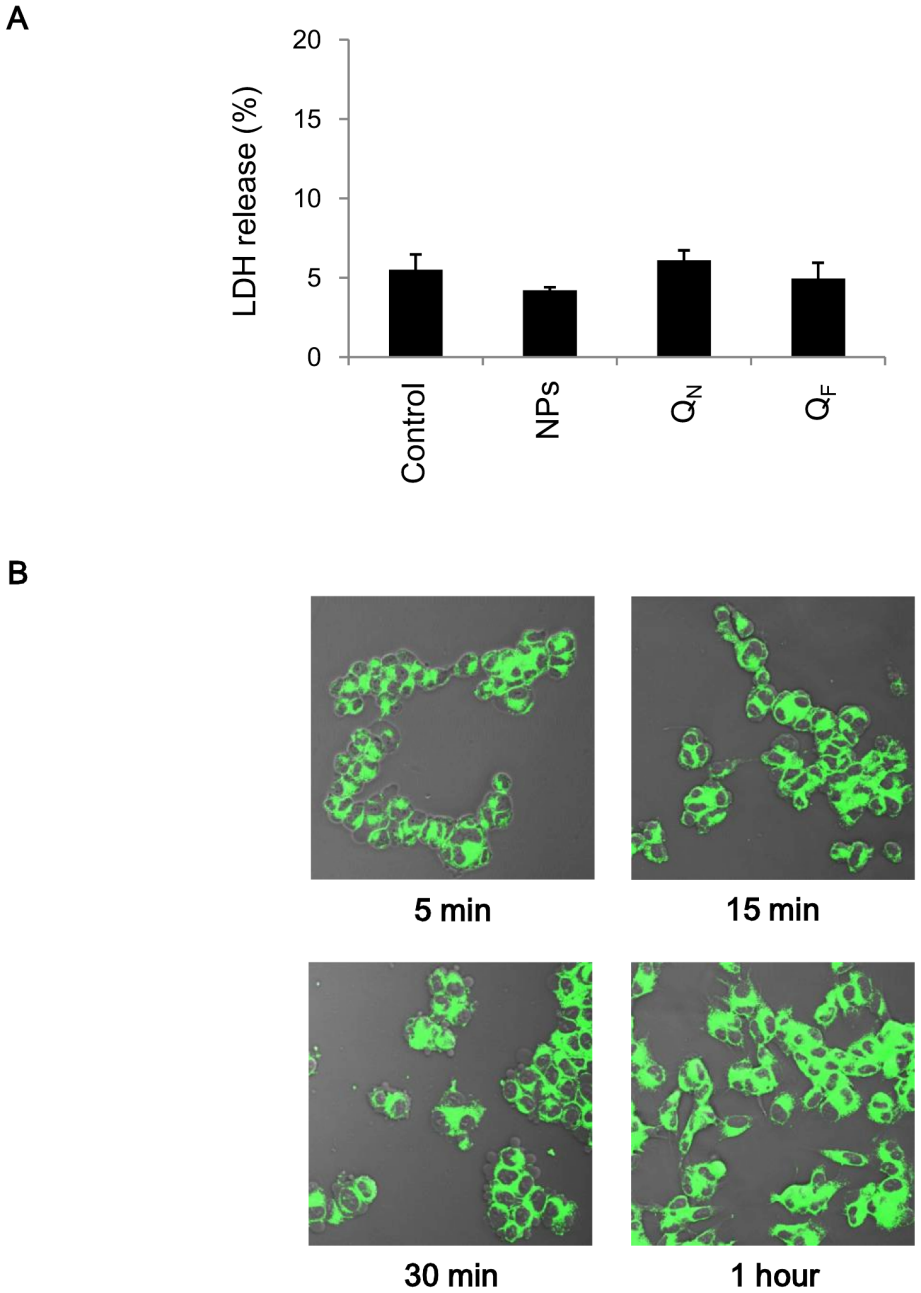
Cell viability and cell uptake of β-CD-dodecylcarbonate nanoparticles. A) SH-SY5Y cells were incubated with β-CD-dodecylcarbonate nanoparticles with (Q_N_) or without (NPs) being loaded with quercetin (5 µM). Some cells were treated with 5 µM quercetin alone (Q_F_). Untreated cells (Control) were taken as controls. Cell viability was measured in terms of release of the enzyme lactate dehydrogenase (LDH), as described in the Materials and Methods section. Data represent the mean values ± SD of three different experiments. B) SH-SY5Y cells were incubated with fluorescent coumarin 6-β-CD-dodecylcarbonate nanoparticles for the times indicated and then analyzed by confocal laser scanning microscopy (40X/0.75).

Before performing our cellular experiments, we tested whether this type of nanoparticle, with or without being loaded with quercetin, is cytotoxic and whether blank nanoparticles (not loaded with quercetin) can be taken up by SH-SY5Y cells. The nanoparticles were found to be non-cytotoxic. Cell viability was measured in terms of release of LDH: neither blank nanoparticles (NPs) nor quercetin-loaded nanoparticles (Q_N_) had any effect on viability of SH-SY5Y cells (Figure 2A). The cytotoxic effect of free quercetin was also tested (Q_F_) and no difference was observed compared with control cells. Fluorescent β-CD-dodecylcarbonate nanoparticles were taken up by the cells in a time-dependent-manner, to a detectable extent already after 5 min of cell incubation, with a maximum after 1 h (Figure 2B). They appear to accumulate in a perinuclear compartment. This would indicate that this carrier system might be useful for drug delivery into neuronal cells. No intracellular fluorescence was detected in control cells that had not been exposed to the fluorescent nanoparticles (data not shown). Some details of the physicochemical characterization (average diameters, polydispersity indices and zeta potentials) of the blank nanoparticles, quercetin-loaded nanoparticles, and β-coumarin-loaded nanoparticles that were used in this research are also reported (Table 1).

**Table 1:** Average diameter, polydispersity index and zeta potential of the nanoparticle formulations.

Nanoparticle types	Average diameter (nm)	Polydispersity index	Zeta potential (mV)
blank	197±8.5	0.08±0.02	−30.2±2.4
quercetin-loaded	214.8±5.6	0.08±0.02	−26.5±1.5
β-coumarin-loaded	210.5±6.2	0.09±0.02	−25.7±1.2

**Figure 3:**
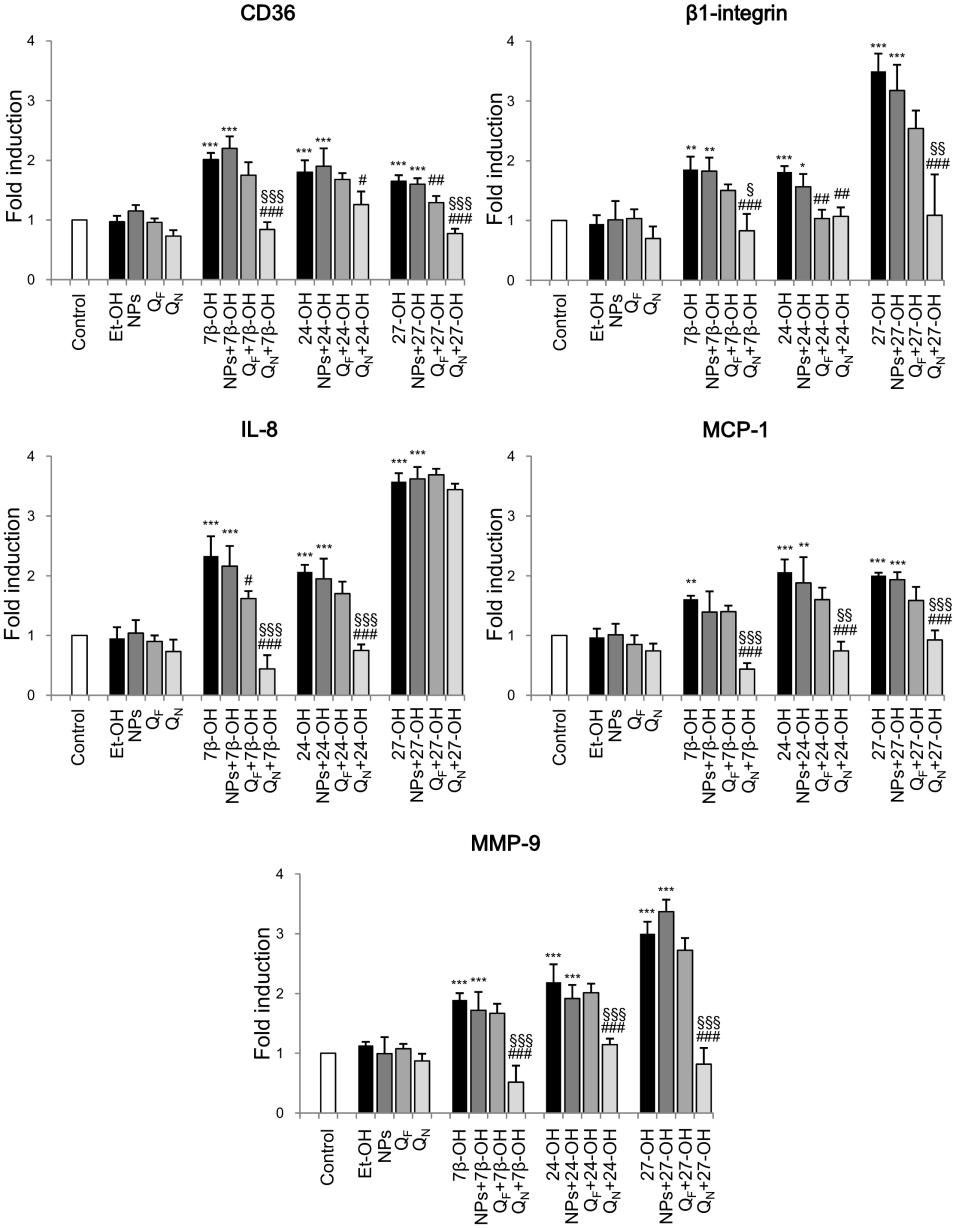
Protection exerted by quercetin-loaded nanoparticles on CD36 and β1-integrin, IL-8, MCP-1, and MMP-9 expression induced by oxysterols. Gene expression was quantified by real-time RT-PCR in SH-SY5Y cells treated for 6 h with 5 µM 7β-hydroxycholesterol (7β-OH), 24-hydroxycholesterol (24-OH), 27-hydroxycholesterol (27-OH). Some cells were pretreated for 1 h with 5 µM free quercetin (Q_F_) or with 5 µM quercetin loaded into nanoparticles (Q_N_) before oxysterol treatment. Untreated cells (Control) were taken as controls, and cells treated with 31.2 mM ethanol (Et-OH) as solvent controls. Cells supplemented with blank nanoparticles (NPs) or with blank nanoparticles plus oxysterols, were taken as internal controls. Data, normalized to β_2_-microglobulin, are expressed as mean values ± SD of five different experiments. *P<0.05, **P<0.01, and ***P<0.001 vs. control; #P<0.05, ##P<0.01, and ###P<0.001 vs. the specific oxysterol; §P<0.05, §§P<0.01, and §§§P<0.001 vs. Q_F_+ specific oxysterol.

**Figure 4:**
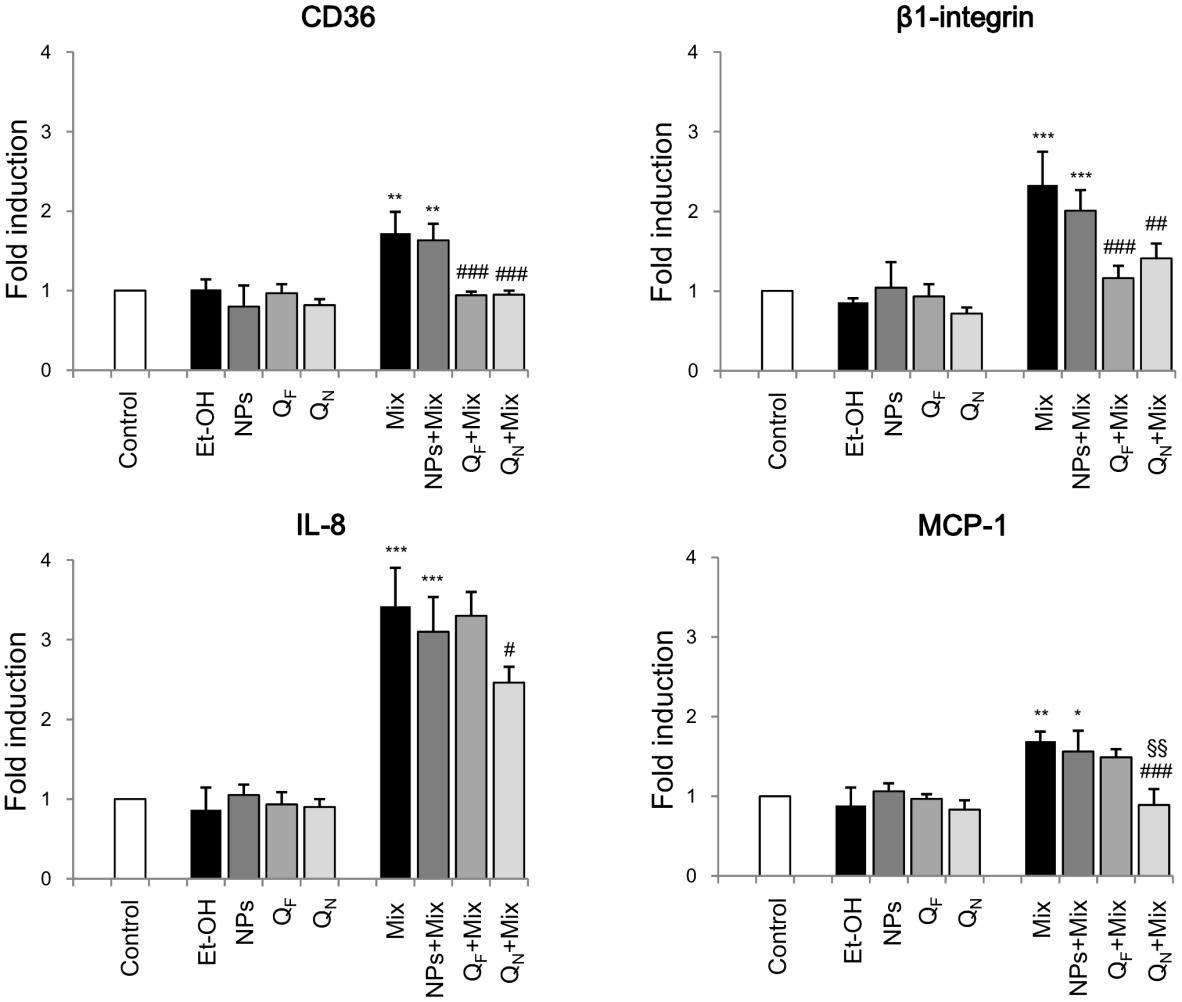
Protection exerted by quercetin-loaded nanoparticles on CD36 and β1-integrin, IL-8, and MCP-1 expression induced by the oxysterol mixture. Gene expression was quantified by real-time RT-PCR in SH-SY5Y cells treated for 6 h with 15 µM oxysterol mixture (Mix). Some cells were pretreated for 1 h with 5 µM free quercetin (Q_F_) or with 5 µM quercetin loaded into nanoparticles (Q_N_) before oxysterol treatment. Untreated cells (Control) were taken as controls, and cells treated with 93.6 mM ethanol (Et-OH) as solvent controls. Cells supplemented with blank nanoparticles (NPs) or with blank nanoparticles plus oxysterol mixture were taken as internal controls. Data, normalized to β_2_-microglobulin, are expressed as mean values ± SD of five different experiments. *P<0.05, **P<0.01, and ***P<0.001 vs. control; #P<0.05, ##P<0.01, and ###P<0.001 vs. the specific oxysterol; §§P<0.01 vs. Q_F_+ specific oxysterol.

The cells were therefore then preincubated with Q_F_ or Q_N_ for 1 h and then with the oxysterols for 6 h. As already reported in Figure 1, expression of the inflammatory mediators up-regulated by oxysterols was investigated by real time RT-PCR. In SH-SY5Y cells, a marked and statistically-significant increase of the expression of the molecules considered was evident after incubation with any of the three oxysterols or with the oxysterol mixture compared with controls (Figure 3 and 4). Cell pretreatment with Q_F_ did not appreciably prevent expression increase of these inflammatory molecules induced by the three oxysterols (Figure 3). Of great interest, conversely, cell pretreatment with Q_N_ produced a clear and statistically-significant down-regulation of these molecules (Figure 3). A similar set of data was obtained when cells were pretreated with Q_N_ and then incubated for 6 h with the oxysterol mixture compared with Q_F_ pretreated cells (Figure 4). MMP-9 gene was not taken into consideration because its expression was not significantly up-regulated by the oxysterol mixture (see Figure 1). In all experiments, cells treated with ethanol (Et-OH) or with blank nanoparticles (NPs) remained unaffected (Figure 3 and 4). Moreover, cell pretreatment with NPs did not interfere with the oxysterols, showing that NPs did not affect the induction of the inflammatory molecule expression induced by the oxysterols themselves (Figure 3 and 4).

### Effects of oxysterols on TLR4 and prevention of its up-regulation by Q_N_ cell pretreatment

**Figure 5:**
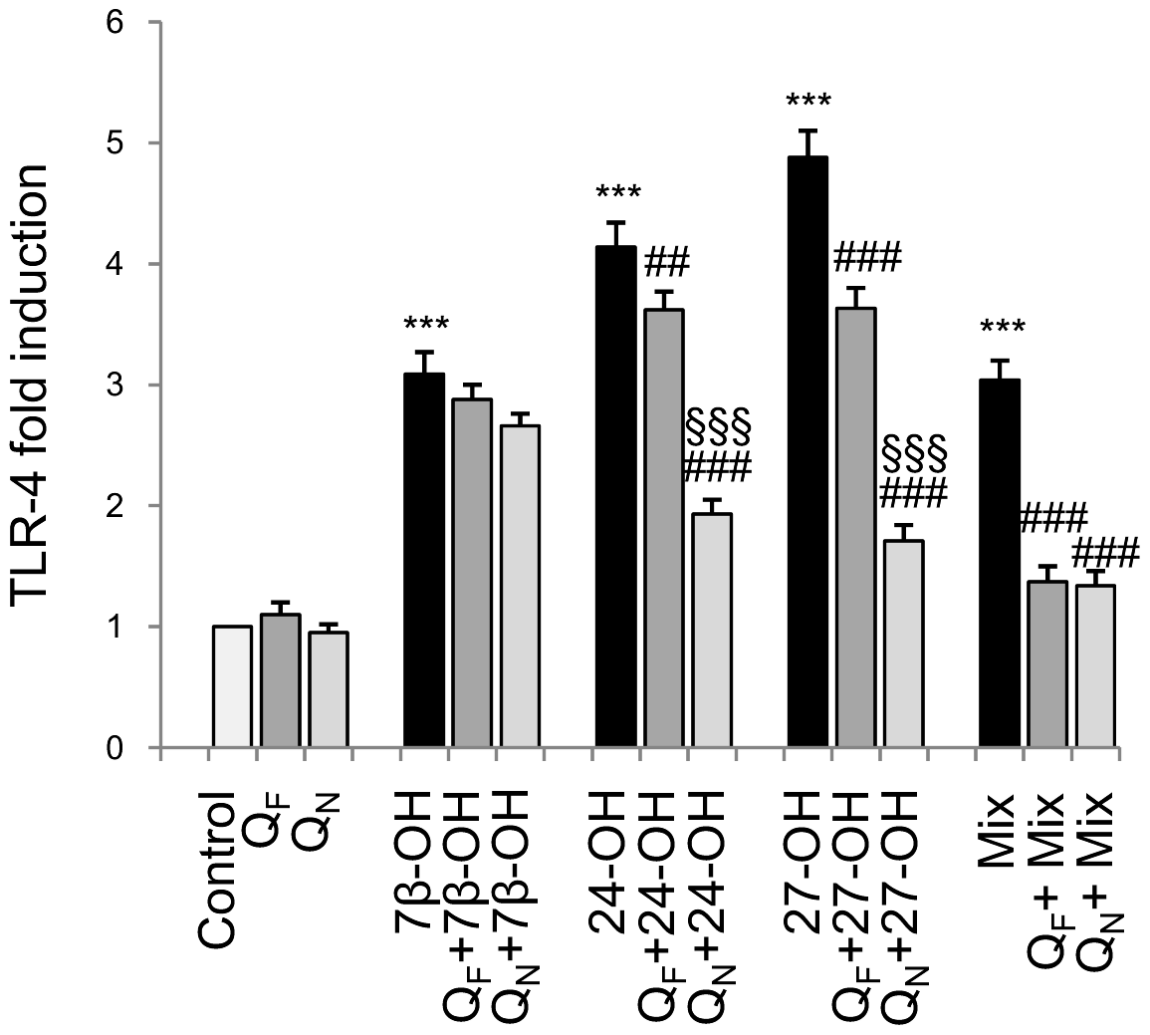
Protective effect of quercetin-loaded nanoparticles on TLR-4 expression induced by oxysterols. Gene expression was quantified by real-time RT-PCR in SH-SY5Y cells treated for 3 h with 5 µM 7β-hydroxycholesterol (7β-OH), 24-hydroxycholesterol (24-OH), 27-hydroxycholesterol (27-OH), or 15 µM oxysterol mixture (Mix). Some cells were pretreated for 1 h with 5 µM free quercetin (Q_F_) or with 5 µM quercetin loaded into nanoparticles (Q_N_) before oxysterol treatment. Untreated cells (Control) were taken as controls. Data, normalized to β_2_-microglobulin, are expressed as mean values ± SD of three different experiments. ***P<0.001 vs. control; ##P<0.01 and ###P<0.001 vs. specific oxysterol; §§§P<0.001 vs. Q_F_+ specific oxysterol.

Up-regulation of TLR4 can contribute to neuroinflammation by amplifying pro-inflammatory cytokine and chemokine release [8], [56]. To determine whether oxysterols might increase TLR4 expression, SH-SY5Y cells were incubated with each individual oxysterol (5 µM) or with a mixture (15 µM) of the three for 3 h. Expression of TLR4 was greatly stimulated, but cell pretreatment (1 h) with 5 µM Q_N_ significantly improved down-regulation of TLR4, in particular in cells treated with 24-OH or 27-OH, compared with cell pretreatment (1 h) with 5 µM Q_F_ (Figure 5), pointing to the efficacy of this drug delivery system.

### Effects of oxysterols on COX-2 and mPGES-1: down-regulation by Q_N_ cell pretreatment

**Figure 6:**
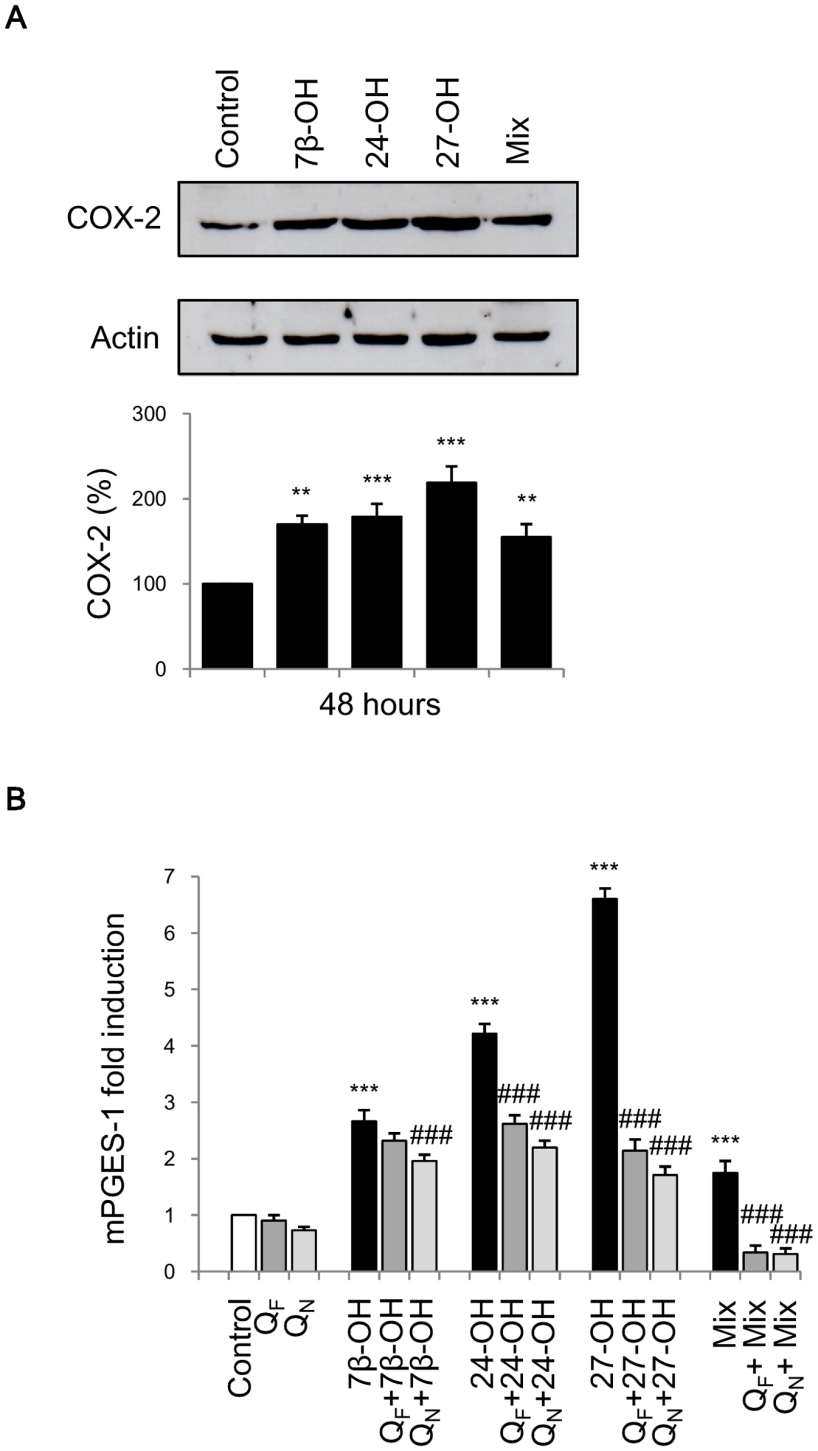
Effect of oxysterols on COX-2 synthesis and mPGES-1 expression. (A) SH-SY5Y cells were treated for 48 h with 5 µM 7β-hydroxycholesterol (7β-OH), 24-hydroxycholesterol (24-OH), 27-hydroxycholesterol (27-OH) or 15 µM oxysterol mixture (Mix). Untreated cells (Control) were taken as controls. COX-2 levels were analyzed by Western blotting. Top: blot representative of three experiments. Bottom: histogram representing mean values ± SD of three experiments. COX-2 densitometric measurements were normalized against the corresponding actin levels and expressed as percentages of control value.**P<0.01 and ***P<0.001 vs. control. (B) mPGES-1 expression was quantified by real-time RT-PCR in SH-SY5Y cells treated for 6 h with 5 µM 7β-OH, 24-OH, 27-OH or 15 µM oxysterol mixture. Some cells were pretreated for 1 h with 5 µM free quercetin (Q_F_) or with 5 µM quercetin loaded into nanoparticles (Q_N_) before oxysterol treatment. Untreated cells (Control) were taken as controls. Data, normalized to β_2_-microglobulin, are expressed as mean values ± SD of three different experiments. ***P<0.001 vs. control; ###P<0.001 vs. specific oxysterol.

Since COXs are up-regulated in many inflammatory disorders including AD [57], to investigate whether oxysterols might be capable of synthesizing the isoform COX-2 in response to inflammatory mediators release, cells were incubated with the oxysterols for 48 h. At the end of the experiment, a significant increase of COX-2 protein levels was observed in SH-SY5Y cells incubated with the individual oxysterols (5 µM) and in those treated with the oxysterol mixture (15 µM) (Figure 6A). Again, 24-OH and 27-OH appear to be the oxysterols contributing most to neuroinflammation.

Besides COX-2, also mPGES-1 is up-regulated during the neuroinflammatory response, with subsequent production of PGE2, which has inflammatory action that impairs brain function. A significant increase of mPGES-1 expression has been observed in cells incubated for 6 h with any of the three oxysterols (Figure 6B). Cell pretreatment with 5 µM Q_F_ significantly reduced the expression of mPGES-1 but cell pretreatment with 5 µM Q_N_ showed a greater reduction of the enzyme expression (Figure 6B).

## Discussion

Neuroinflammation, whether as cause or consequence of AD, plays a central role in the pathogenesis of this neurodegenerative disease [4]–[8]. The importance of neuroinflammatory processes has been emphasized during the past decade, as intensive investigations have examined pro-inflammatory mediators and free radical-mediated oxidative stress, both of which potentially contribute to further neuronal dysfunction and cell death, as well as to glial cell activation in the brain of AD patients [14]–[18]. Although neuroinflammation principally involves activating the microglia and astrocytes, it has recently been suggested that neurons themselves may react and contribute to the chronic neuroinflammatory changes in AD, by serving as a source of inflammatory mediators [6].

Of note, epidemiological and biochemical data also seem to suggest that there may be a mechanistic link among altered brain cholesterol metabolism, neuroinflammation and AD pathogenesis [22], [23], [26]–[28]. The idea that oxysterols, a class of cholesterol oxidation products, might be the missing link between altered brain cholesterol metabolism and AD has gained increasing support as a growing body of evidence suggests the involvement of 24-OH and 27-OH in neurotoxicity [23]. In our recent studies, we found that the three oxysterols 24-OH, 27-OH and 7β-OH, and 4-hydroxynonenal (HNE) (the most reactive end-product of lipid peroxidation, which contributes to neuron dysfunction and death), strongly enhance the binding and accumulation of Aβ_1-42_ on membranes of human differentiated neuronal cell lines (SK-N-BE and NT-2) and of human dental-pulp neuron-like cells (DPNLC), respectively. The mechanism involves the marked increase of the availability of the multireceptor complex CD36/β1-integrin/CD47. Interesting findings of these studies are that only 24-OH and HNE significantly potentiate the neurotoxic action of Aβ_1–42_ on these cells by locally increasing ROS steady-state levels [58], [59]. Moreover, in neuronal SK-N-BE and NT-2 cells, 24-OH and 27-OH have been shown to enhance expression and activity of the β- and γ-secretases of the amyloidogenic pathway of amyloid precursor protein processing, leading to increased Aβ synthesis and accumulation in those cells [60]. Further, as regards the potential neurotoxicity of oxysterols, 24-OH has also been shown to cause cell death when added to undifferentiated (50 µM) and differentiated (25–50 µM) SH-SY5Y cells, an affect that was mediated by increased generation of free radicals [61], [62]. The neurotoxicity of 24-OH was partially prevented by the free radical scavenger vitamin E (α-tocopherol) and by estradiol-17β [62]. Moreover, 7β-OH has been found to be neurotoxic at nanomolar concentrations in cultured rat hippocampal neuronal cells, and may therefore contribute to Aβ-related neurodegeneration in the brain of AD patients [25]. Another oxysterol that has been found responsible for necrotic cell death of SH-SY5Y cells is 7α-hydroperoxycholesterol, which might derive from the auto-oxidation of cellular cholesterol, released during neurodegeneration [63]; a further possibility is 7-ketocholesterol [64]. Additionally, in agreement with Gamba and colleagues [60] 24-OH has been shown to enhance the neurotoxic effect of the Aβ_1–42_ peptide in the human differentiated neuroblastoma cell line MSN, as well as augmenting ROS generation [65]. There is also mounting evidence that treatment with oxysterols enhances the release of a number of inflammatory molecules in a wide variety of cell types [45]–[50].

Although oxysterols have been studied for their involvement in oxidative stress and inflammatory processes, and in the subsequent cell death during AD progression [23], it is now emerging that they play a role as ligands (e.g. 24-OH and 27-OH) for liver X receptors (LXRs), transcription factors that regulate an array of genes, among them the genes involved in cholesterol efflux and metabolism [66], [67]. Indeed, astrocytes are sensitive to 24-OH-mediated up-regulation of the LXR-responsive genes involved in cholesterol efflux: ATP-binding cassette transporter A1 and G1 (ABCA1 and ABCG1) and apolipoprotein E [68]. Of note, contrary to Gamba and colleagues [60], 27-OH, as an LXR ligand, has been shown to significantly exert anti-amyloidogenic effects, by reducing Aβ peptide generation from primary human neurons, in turn by up-regulating LXR responsive genes [69]. Recent in vitro evidence also suggests that LXR activation by 24-OH and 27-OH may contribute to decreasing the Aβ peptide influx across the BBB, with involvement of the ABCB1 transporter, leading to protection from peripheral Aβ entry [70]. Conversely, treatment of brain pericytes with 24-OH caused an increase in ABCA1 expression correlated with an increase of cholesterol efflux, but 24-OH treatment was found not to reduce the ability of the pericytes to accumulate Aβ in the cells [71]. The clearance of Aβ also seems to be mediated through microglia-induced phagocytosis, which is dependent on LXR activation [72].

Of note, LXR activation not only affects gene regulation of cholesterol homeostasis, and Aβ peptide transport and clearance, but also inflammation in the brain. Studies have demonstrated that LXR activation inhibits AD-related inflammatory responses and inflammatory gene expression, owing to LXR's ability to functionally inactivate the promoters of pro-inflammatory genes and of nuclear factor-κB (NF-κB) [73]–[78]. Moreover, LXR activation may prevent an inflammatory response by indirectly supporting repression of TLR target gene activation, which may modulate inflammatory signaling via several routes.

However, although LXR-activating oxysterols might reduce membrane cholesterol content and inflammation, they can also act by activating opposing pathways and inducing expression of inflammation markers independently of LXRs in endothelial cells [46].

In this study we report clear evidence that the oxysterols potentially involved in AD pathogenesis markedly enhance pro-inflammatory mediator expression, which plays a critical role in mediating AD-associated changes, also by driving a self-sustaining cycle that exacerbates neuron loss. In this connection, we found that 24-OH and 27-OH, but also 7β-OH, induced expression of IL-8, MCP-1, β1-integrin, CD36, and MMP-9 in human neuroblastoma SH-SY5Y cells. The chemokines IL-8 and MCP-1 are important mediators for both microglia and astrocyte recruitment and activation as well as leukocyte infiltration around the areas of neuroinflammation [79], [80]. The adhesion molecule β1-integrin also plays a critical role in regulating leukocyte migration through ECM to the site of inflammation, by mediating cell-cell interactions and by connecting the ECM molecules to the cellular cytoskeleton [81]. Moreover, MMP-9 has been identified in neuroinflammation and, of note, its expression is regulated, among other factors, by cytokines [82], [83]. In addition, the scavenger receptor CD36 plays a fundamental role in binding the Aβ peptide [58] as well as in cerebrovascular oxidative stress and neurovascular dysfunction induced by Aβ, promoting inflammation [84]. Of note, some of the pro-inflammatory effects of CD36 have been attributed to its association with TLRs heterodimers (TLR2/1, TLR2/6, or TLR4/6) as co-receptor, leading to NF-κB activation and pro-inflammatory gene expression. In particular, the pro-inflammatory signaling of Aβ depends on its interaction with CD36 which induce the downstream signaling cascades required for TLR4/6 activation [85]. It is thus clear that TLR4 activation contributes to neuroinflammation by amplifying the release of pro-inflammatory mediators [8], [51].

On this basis, we here show that oxysterols can stimulate TLR4 expression, potentially leading to an increase of inflammatory molecule release in SH-SY5Y cells. Other research groups have reported the hypothesis that oxysterols might promote inflammation via TLR2/4 activation [86], [87]. Moreover, we found that oxysterols increase the levels of COX-2, as well as expression of mPGES-1, both of which are stimulated by cytokine and chemokine release, with subsequent production of prostaglandin E2. These molecular mechanisms, induced by oxysterols, thus play a fundamental role in the neuroinflammatory changes in AD.

To highlight the inflammatory actions of these oxysterols, we preincubated the cells with the bioflavonoid quercetin, which, as do the other polyphenols, exerts neuroprotective action. The polyphenols attenuate or prevent oxidative stress and inflammatory changes, thanks to their anti-oxidant, anti-inflammatory, and anti-amyloidogenic activities [30]–[35]. Because quercetin, like the other polyphenols, cannot easily pass through the BBB, and like most of them has limited bioavailability and is extensively metabolized [39], [40], to investigate a new delivery form that would improve its bioavailability, and consequently its anti-inflammatory activity, quercetin was delivered to cells loaded into β-CD-dodecylcarbonate nanoparticles. The anti-inflammatory effect of quercetin-loaded nanoparticles was markedly stronger than that of free quercetin. The ability of quercetin-loaded nanoparticles to prevent or reduce inflammatory molecule expression is also been demonstrated by the fact that it can down-regulate the TLR4 and COX-2 signaling cascades.

Although further studies are required to elucidate the precise mechanisms and action of quercetin-loaded nanoparticles, the present findings support the hypothesis that this drug delivery system might be a potential new therapeutic tool, that could increase quercetin's neuroprotective effects by improving its permeation across the BBB into the brain, and its bioavailability, thus its ability to reach target cells.

## References

[1] Querfurth HW, LaFerla FM (2010) Alzheimer's disease. N Engl J Med 362: 329–44. 20107219 10.1056/NEJMra0909142

[2] Chopra K, Misra S, Kuhad A (2011) Neurobiological aspects of Alzheimer's disease. Expert Opin Ther Targets 15: 535–555. 21314231 10.1517/14728222.2011.557363

[3] Quintanilla RA, Orellana JA, von Bernhardi R (2012) Understanding risk factors for Alzheimer's disease: interplay of neuroinflammation, connexin-based communication and oxidative stress. Arch Med Res 43: 632–644. 23142264 10.1016/j.arcmed.2012.10.016

[4] Akiyama H, Barger S, Barnum S, Bradt B, Bauer J, et al (2000) Inflammation and Alzheimer's disease. Neurobiol Aging 21: 383–421. 10858586 10.1016/s0197-4580(00)00124-x PMC3887148

[5] Wyss-Coray T (2006) Inflammation in Alzheimer disease: driving force, bystander or beneficial response? Nat Med 12: 1005–1015. 16960575 10.1038/nm1484

[6] Heneka MT, O'Banion MK (2007) Inflammatory processes in Alzheimer's disease. J Neuroimmunol 184: 69–91. 17222916 10.1016/j.jneuroim.2006.11.017

[7] Holmes C, Butchart J (2011) Systemic inflammation and Alzheimer's disease. Biochem Soc Trans 39: 898–901. 21787320 10.1042/BST0390898

[8] Lyman M, Lloyd DG, Ji X, Vizcaychipi MP, Ma D (2013) Neuroinflammation: The role and consequences. Neurosci Res doi: 10.1016/j.neures.2013.10.004 10.1016/j.neures.2013.10.004 24144733

[9] Holmes C (2013) Review: systemic inflammation and Alzheimer's disease. Neuropathol Appl Neurobiol 39: 51–68. 23046210 10.1111/j.1365-2990.2012.01307.x

[10] Perry VH, Nicoll JA, Holmes C (2010) Microglia in neurodegenerative disease. Nat Rev Neurol 6: 193–201. 20234358 10.1038/nrneurol.2010.17

[11] McGeer EG, McGeer PL (2010) Neuroinflammation in Alzheimer's disease and mild cognitive impairment: a field in its infancy. J Alzheimers Dis 19: 355–361. 20061650 10.3233/JAD-2010-1219

[12] Popescu BO, Toescu EC, Popescu LM, Bajenaru O, Muresanu DF, et al (2009) Blood-brain barrier alterations in ageing and dementia. J Neurol Sci 283: 99–106. 19264328 10.1016/j.jns.2009.02.321

[13] Leoni V, Masterman T, Patel P, Meaney S, Diczfalusy U, et al (2003) Side chain oxidized oxysterols in cerebrospinal fluid and the integrity of blood-brain and blood-cerebrospinal fluid barriers. J Lipid Res 44: 793–799. 12562838 10.1194/jlr.M200434-JLR200

[14] von Bernhardi R (2007) Glial cell dysregulation: a new perspective on Alzheimer disease. Neurotox Res 12: 215–232. 18201950 10.1007/BF03033906

[15] Heneka MT, O'Banion MK, Terwel D, Kummer MP (2010) Neuroinflammatory processes in Alzheimer's disease. J Neural Transm 17: 919–947. 10.1007/s00702-010-0438-z 20632195

[16] Glass CK, Saijo K, Winner B, Marchetto MC, Gage FH (2010) Mechanisms underlying inflammation in neurodegeneration. Cell 140: 918–934. 20303880 10.1016/j.cell.2010.02.016 PMC2873093

[17] Bhamra MS, Ashton NJ (2012) Finding a pathological diagnosis for Alzheimer's disease: are inflammatory molecules the answer? Electrophoresis 33: 3598–3607. 23161254 10.1002/elps.201200161

[18] Azizi G, Mirshafiey A (2012) The potential role of proinflammatory and antiinflammatory cytokines in Alzheimer disease pathogenesis, Immunopharmacol Immunotoxicol. 34: 881–895. 10.3109/08923973.2012.705292 22970774

[19] Colton C, Wilcock DM (2010) Assessing activation states in microglia. CNS Neurol Disord Drug Targets 9: 174–191. 20205642 10.2174/187152710791012053

[20] Weitz TM, Town T (2012) Microglia in Alzheimer's Disease: It's All About Context. Int J Alzheimers Dis 2012: 314185. 22779026 10.1155/2012/314185 PMC3388286

[21] Johnston H, Boutin H, Allan SM (2011) Assessing the contribution of inflammation in models of Alzheimer's disease. Biochem Soc Trans 39: 886–890. 21787318 10.1042/BST0390886

[22] Björkhem I, Cedazo-Minguez A, Leoni V, Meaney S (2009) Oxysterols and neurodegenerative diseases. Mol Aspects Med 30: 171–179. 19248803 10.1016/j.mam.2009.02.001

[23] Gamba P, Testa G, Sottero B, Gargiulo S, Poli G, et al (2012) The link between altered cholesterol metabolism and Alzheimer's disease. Ann N Y Acad Sci 1259: 54–64. 22758637 10.1111/j.1749-6632.2012.06513.x

[24] Björkhem I, Heverin M, Leoni V, Meaney S, Diczfalusy U (2006) Oxysterols and Alzheimer's disease. Acta Neurol Scand Suppl 185: 43–49. 10.1111/j.1600-0404.2006.00684.x 16866910

[25] Nelson TJ, Alkon DL (2005) Oxidation of cholesterol by amyloid precursor protein and beta-amyloid peptide. J Biol Chem 280: 7377–7387. 15591071 10.1074/jbc.M409071200

[26] Vaya J, Schipper HM (2007) Oxysterols, cholesterol homeostasis, and Alzheimer disease. J Neurochem 102: 1727–1737. 17573819 10.1111/j.1471-4159.2007.04689.x

[27] Leoni V, Caccia C (2011) Oxysterols as biomarkers in neurodegenerative diseases. Chem Phys Lipids 164: 515–524. 21515244 10.1016/j.chemphyslip.2011.04.002

[28] Puglielli L, Tanzi RE, Kovacs DM (2003) Alzheimer's disease: the cholesterol connection. Nat Neurosci 6: 345–351. 12658281 10.1038/nn0403-345

[29] Faria A, Pestana D, Teixeira D, Azevedo J, De Freitas V, et al (2010) Flavonoid transport across RBE4 cells: A blood-brain barrier model. Cell Mol Biol Lett 15: 234–241. 20140760 10.2478/s11658-010-0006-4 PMC6275689

[30] Vafeiadou K, Vauzour D, Spencer JP (2007) Neuroinflammation and its modulation by flavonoids. Endocr Metab Immune Disord Drug Targets 7: 211–224. 17897048 10.2174/187153007781662521

[31] Ho L, Pasinetti GM (2010) Polyphenolic compounds for treating neurodegenerative disorders involving protein misfolding. Expert Rev Proteomics 7: 579–589. 20653511 10.1586/epr.10.69

[32] Mandel SA, Weinreb O, Amit T, Youdim MB (2012) Molecular mechanisms of the neuroprotective/neurorescue action of multi-target green tea polyphenols. Front Biosci (Schol Ed) 4: 581–598. 22202078 10.2741/S286

[33] Jayasena T, Poljak A, Smythe G, Braidy N, Münch G, et al (2013) The role of polyphenols in the modulation of sirtuins and other pathways involved in Alzheimer's disease. Ageing Res Rev 12: 867–883. 23831960 10.1016/j.arr.2013.06.003

[34] Bhullar KS, Rupasinghe HP (2013) Polyphenols: multipotent therapeutic agents in neurodegenerative diseases. Oxid Med Cell Longev 2013: 891748. 23840922 10.1155/2013/891748 PMC3690243

[35] Dajas F, Andrés AC, Florencia A, Carolina E, Felicia RM (2013) Neuroprotective actions of flavones and flavonols: mechanisms and relationship to flavonoid structural features. Cent Nerv Syst Agents Med Chem 13: 30–35. 23092407 10.2174/1871524911313010005

[36] Bischoff SC (2008) Quercetin: potentials in the prevention and therapy of disease. Curr Opin Clin Nutr Metab Care 11: 733–740. 18827577 10.1097/MCO.0b013e32831394b8

[37] Bureau G, Longpré F, Martinoli MG (2008) Resveratrol and quercetin, two natural polyphenols, reduce apoptotic neuronal cell death induced by neuroinflammation. J Neurosci Res 86: 403–410. 17929310 10.1002/jnr.21503

[38] Ansari MA, Abdul HM, Joshi G, Opii WO, Butterfield DA (2009) Protective effect of quercetin in primary neurons against Abeta(1–42): relevance to Alzheimer's disease. J Nutr Biochem 20: 269–275. 18602817 10.1016/j.jnutbio.2008.03.002 PMC2737260

[39] Leonarduzzi G, Testa G, Sottero B, Gamba P, Poli G (2010) Design and development of nanovehicle-based delivery systems for preventive or therapeutic supplementation with flavonoids. Curr Med Chem 17: 74–95. 19941477 10.2174/092986710789957760

[40] Scheepens A, Tan K, Paxton JW (2010) Improving the oral bioavailability of beneficial polyphenols through designed synergies. Genes Nutr 5: 75–87. 19841960 10.1007/s12263-009-0148-z PMC2820202

[41] Sahni JK, Doggui S, Ali J, Baboota S, Dao L, et al (2011) Neurotherapeutic applications of nanoparticles in Alzheimer's disease. J Control Release 152: 208–231. 21134407 10.1016/j.jconrel.2010.11.033

[42] Doggui S, Dao L, Ramassamy C (2012) Potential of drug-loaded nanoparticles for Alzheimer's disease: diagnosis, prevention and treatment. Ther Deliv 3: 1025–1027. 23035588 10.4155/tde.12.84

[43] Ghosh A, Mandal AK, Sarkar S, Panda S, Das N (2009) Nanoencapsulation of quercetin enhances its dietary efficacy in combating arsenic-induced oxidative damage in liver and brain of rats. Life Sci 84: 75–80. 19036345 10.1016/j.lfs.2008.11.001

[44] Dhawan S, Kapil R, Singh B (2011) Formulation development and systematic optimization of solid lipid nanoparticles of quercetin for improved brain delivery. J Pharm Pharmacol 63: 342–351. 21749381 10.1111/j.2042-7158.2010.01225.x

[45] Prunet C, Montange T, Véjux A, Laubriet A, Rohmer JF, et al (2006) Multiplexed flow cytometric analyses of pro- and anti-inflammatory cytokines in the culture media of oxysterol-treated human monocytic cells and in the sera of atherosclerotic patients. Cytometry A 69: 359–373. 16604541 10.1002/cyto.a.20272

[46] Morello F, Saglio E, Noghero A, Schiavone D, Williams TA, et al (2009) LXR-activating oxysterols induce the expression of inflammatory markers in endothelial cells through LXR-independent mechanisms. Atherosclerosis 207: 38–44. 19426978 10.1016/j.atherosclerosis.2009.04.001

[47] Mascia C, Maina M, Chiarpotto E, Leonarduzzi G, Poli G, et al (2010) Proinflammatory effect of cholesterol and its oxidation products on CaCo-2 human enterocyte-like cells: effective protection by epigallocatechin-3-gallate. Free Radic Biol Med 49: 2049–2057. 20923702 10.1016/j.freeradbiomed.2010.09.033

[48] Dugas B, Charbonnier S, Baarine M, Ragot K, Delmas D, et al (2010) Effects of oxysterols on cell viability, inflammatory cytokines, VEGF, and reactive oxygen species production on human retinal cells: cytoprotective effects and prevention of VEGF secretion by resveratrol. Eur J Nutr 49: 435–446. 20339855 10.1007/s00394-010-0102-2

[49] Sottero B, Gamba P, Gargiulo S, Leonarduzzi G, Poli G (2009) Cholesterol oxidation products and disease: an emerging topic of interest in medicinal chemistry. Curr Med Chem 16: 685–705. 19199932 10.2174/092986709787458353

[50] Vejux A, Lizard G (2009) Cytotoxic effects of oxysterols associated with human diseases: Induction of cell death (apoptosis and/or oncosis), oxidative and inflammatory activities, and phospholipidosis. Mol Aspects Med 30: 153–170. 19248805 10.1016/j.mam.2009.02.006

[51] Cavalli R, Trotta F, Trotta M, Pastero L, Aquilano D (2007) Effect of alkylcarbonate of γ-cyclodextrins with different chain lengths on drug complexation and release characteristics. Int J Pharm 339: 197–204. 17418510 10.1016/j.ijpharm.2007.03.001

[52] Cavalli R, Trotta F, Carlotti ME, Possetti B, Trotta M (2007) Nanoparticles derived from amphiphilicγ-cyclodextrins. J Incl Phenom Macrocycl Chem 57: 657–661.

[53] Cavalli R, Donalisio M, Civra A, Ferruti P, Ranucci E, et al (2009) Enhanced antiviral activity of acyclovir loaded into β- cyclodextrin-poly(4-acryloylmorpholine)conjugate nanoparticles. J Control Release 137: 116–122. 19361545 10.1016/j.jconrel.2009.04.004

[54] Hunter RJ (1981) Zeta potential in colloid science. Principles and applications. Academic Press London.

[55] Livak JK, Schmittgen TD (2001) Analysis of relative gene expression data using real-time quantitative PCR and the 2(-Delta Delta C(T)). Method Methods 25: 402–408. 11846609 10.1006/meth.2001.1262

[56] Drouin-Ouellet J, Cicchetti F (2012) Inflammation and neurodegeneration: the story ‘retolled’. Trends Pharmacol Sci 33: 542–551. 22944460 10.1016/j.tips.2012.07.002

[57] Hein AM, O'Banion MK (2009) Neuroinflammation and memory: the role of prostaglandins. Mol Neurobiol 40: 15–32. 19365736 10.1007/s12035-009-8066-z PMC3124778

[58] Gamba P, Leonarduzzi G, Tamagno E, Guglielmotto M, Testa G, et al (2011) Interaction between 24-hydroxycholesterol, oxidative stress, and amyloid-β in amplifying neuronal damage in Alzheimer's disease: three partners in crime. Aging Cell 10: 403–417. 21272192 10.1111/j.1474-9726.2011.00681.x

[59] Testa G, Gamba P, Di Scipio F, Sprio AE, Salamone P, et al (2012) Potentiation of amyloid-β peptide neurotoxicity in human dental-pulp neuron-like cells by the membrane lipid peroxidation product 4-hydroxynonenal. Free Radic Biol Med 53: 1708–1717. 22981873 10.1016/j.freeradbiomed.2012.08.581

[60] Gamba P, Guglielmotto M, Testa G, Monteleone D, Zerbinati C, et al.. (2014) Up-regulation of b-amyloidogenesis in neuron-like human cells by both 24- and 27-hydroxycholesterol: protective effect of N-acetyl-cysteine. Aging Cell, doi: 10.1111/acel.12206. 10.1111/acel.12206 PMC4326893 24612036

[61] Kölsch H, Lütjohann D, Tulke A, Björkhem I, Rao ML (1999) The neurotoxic effect of 24-hydroxycholesterol on SH-SY5Y human neuroblastoma cells. Brain Res 818: 171–175. 9914453 10.1016/s0006-8993(98)01274-8

[62] Kölsch H, Ludwig M, Lütjohann D, Rao ML (2001) Neurotoxicity of 24-hydroxycholesterol, an important cholesterol elimination product of the brain, may be prevented by vitamin E and estradiol-17beta. J Neural Transm 108: 475–488. 11475014 10.1007/s007020170068

[63] Kölsch H, Ludwig M, Lütjohann D, Prange W, Rao ML (2000) 7alpha-Hydroperoxycholesterol causes CNS neuronal cell death. Neurochem Int 36: 507–512. 10762087 10.1016/s0197-0186(99)00157-6

[64] Ong WY, Kim JH, He X, Chen P, Farooqui AA, et al (2010) Changes in brain cholesterol metabolome after excitotoxicity. Mol Neurobiol 41: 299–313. 20140539 10.1007/s12035-010-8099-3

[65] Ferrera P, Mercado-Gómez O, Silva-Aguilar M, Valverde M, Arias C (2008) Cholesterol potentiates beta-amyloid-induced toxicity in human neuroblastoma cells: involvement of oxidative stress. Neurochem Res 33: 1509–1517. 18288607 10.1007/s11064-008-9623-y

[66] Zelcer N, Tontonoz P (2006) Liver X receptors as integrators of metabolic and inflammatory signaling. J Clin Invest 116: 607–614. 16511593 10.1172/JCI27883 PMC1386115

[67] Panzenboeck U, Kratzer I, Sovic A, Wintersperger A, Bernhart E, et al (2006) Regulatory effects of synthetic liver X receptor- and peroxisome-proliferator activated receptor agonists on sterol transport pathways in polarized cerebrovascular endothelial cells. Int J Biochem Cell Biol 38: 1314–1329. 16530456 10.1016/j.biocel.2006.01.013

[68] Abildayeva K, Jansen PJ, Hirsch-Reinshagen V, Bloks VW, Bakker AH, et al (2006) 24(S)-hydroxycholesterol participates in a liver X receptor-controlled pathway in astrocytes that regulates apolipoprotein E-mediated cholesterol efflux. J Biol Chem 281: 12799–12808. 16524875 10.1074/jbc.M601019200

[69] Kim WS, Chan SL, Hill AF, Guillemin GJ, Garner B (2009) Impact of 27-hydroxycholesterol on amyloid-beta peptide production and ATP-binding cassette transporter expression in primary human neurons. J Alzheimers Dis 16: 121–131. 19158428 10.3233/JAD-2009-0944

[70] Saint-Pol J, Candela P, Boucau MC, Fenart L, Gosselet F (2013) Oxysterols decrease apical-to-basolateral transport of Aβ peptides via an ABCB1-mediated process in an in vitro Blood-brain barrier model constituted of bovine brain capillary endothelial cells. Brain Res 1517: 1–15. 23603412 10.1016/j.brainres.2013.04.008

[71] Saint-Pol J, Vandenhaute E, Boucau MC, Candela P, Dehouck L, et al (2014) Brain pericytes ABCA1 expression mediates cholesterol efflux but not cellular amyloid-β peptide accumulation. J Alzheimers Dis 30: 489–503. 10.3233/JAD-2012-112090 22433669

[72] Terwel D, Steffensen KR, Verghese PB, Kummer MP, Gustafsson JÅ, et al (2011) Critical role of astroglial apolipoprotein E and liver X receptor-α expression for microglial Aβ phagocytosis. J Neurosci 31: 7049–7059. 21562267 10.1523/JNEUROSCI.6546-10.2011 PMC6703224

[73] Wang L, Schuster GU, Hultenby K, Zhang Q, Andersson S, et al (2002) Liver X receptors in the central nervous system: from lipid homeostasis to neuronal degeneration. Proc Natl Acad Sci USA 15: 13878–13883. 10.1073/pnas.172510899 PMC129791 12368482

[74] Riddell DR, Zhou H, Comery TA, Kouranova E, Lo CF, et al (2007) The LXR agonist TO901317 selectively lowers hippocampal Abeta42 and improves memory in the Tg2576 mouse model of Alzheimer's disease. Mol Cell Neurosci 34: 621–628. 17336088 10.1016/j.mcn.2007.01.011

[75] Zelcer N, Khanlou N, Clare R, Jiang Q, Reed-Geaghan EG, et al (2007) Attenuation of neuroinflammation and Alzheimer's disease pathology by liver x receptors. Proc Natl Acad Sci USA 104: 10601–10606. 17563384 10.1073/pnas.0701096104 PMC1890560

[76] Cao G, Bales KR, DeMattos RB, Paul SM (2007) Liver X receptor-mediated gene regulation and cholesterol homeostasis in brain: relevance to Alzheimer's disease therapeutics. Curr Alzheimer Res 4: 179–184. 17430244 10.2174/156720507780362173

[77] Steffensen KR, Jakobsson T, Gustafsson JÅ (2013) Targeting liver X receptors in inflammation. Expert Opin Ther Targets 17: 977–990. 23738533 10.1517/14728222.2013.806490

[78] Sodhi RK, Singh N (2013) Liver X receptors: emerging therapeutic targets for Alzheimer's disease. Pharmacol Res 72: 45–51. 23542729 10.1016/j.phrs.2013.03.008

[79] Bajetto A, Bonavia R, Barbero S, Florio T, Schettini G (2001) Chemokines and their receptors in the central nervous system. Front Neuroendocrinol 22: 147–184. 11456467 10.1006/frne.2001.0214

[80] Conductier G, Blondeau N, Guyon A, Nahon JL, Rovère C (2010) The role of monocyte chemoattractant protein MCP1/CCL2 in neuroinflammatory diseases. J Neuroimmunol 224: 93–100. 20681057 10.1016/j.jneuroim.2010.05.010

[81] Haanstra KG, Hofman SO, Lopes Estêvão DM, Blezer EL, Bauer J, et al (2013) Antagonizing the α4β1 integrin, but not α4β7, inhibits leukocytic infiltration of the central nervous system in rhesus monkey experimental autoimmune encephalomyelitis. J Immunol 190: 1961–1973. 23365083 10.4049/jimmunol.1202490

[82] Candelario-Jalil E, Yang Y, Rosenberg GA (2009) Diverse roles of matrix metalloproteinases and tissue inhibitors of metalloproteinases in neuroinflammation and cerebral ischemia. Neuroscience 158: 983–994. 18621108 10.1016/j.neuroscience.2008.06.025 PMC3584171

[83] Dafnis I, Tzinia AK, Tsilibary EC, Zannis VI, Chroni A (2012) An apolipoprotein E4 fragment affects matrix metalloproteinase 9, tissue inhibitor of metalloproteinase 1 and cytokine levels in brain cell lines. Neuroscience 210: 21–32. 22445724 10.1016/j.neuroscience.2012.03.013 PMC3358542

[84] Park L, Wang G, Zhou P, Zhou J, Pitstick R, et al (2011) Scavenger receptor CD36 is essential for the cerebrovascular oxidative stress and neurovascular dysfunction induced by amyloid-beta. Proc Natl Acad Sci USA 108: 5063–5068. 21383152 10.1073/pnas.1015413108 PMC3064396

[85] Stewart CR, Stuart LM, Wilkinson K, van Gils JM, Deng J, et al (2010) CD36 ligands promote sterile inflammation through assembly of a Toll-like receptor 4 and 6 heterodimer. Nat Immunol 11: 155–161. 20037584 10.1038/ni.1836 PMC2809046

[86] Chávez-Sánchez L, Madrid-Miller A, Chávez-Rueda K, Legorreta-Haquet MV, Tesoro-Cruz E, et al (2010) Activation of TLR2 and TLR4 by minimally modified low-density lipoprotein in human macrophages and monocytes triggers the inflammatory response. Hum Immunol 71: 737–744. 20472010 10.1016/j.humimm.2010.05.005

[87] Aye IL, Waddell BJ, Mark PJ, Keelan JA (2012) Oxysterols exert proinflammatory effects in placental trophoblasts via TLR4-dependent, cholesterol-sensitive activation of NF-κB. Mol Hum Reprod 18: 341–353. 22238372 10.1093/molehr/gas001

